# Probing the Effective Treatment Thresholds for Alteplase in Acute Ischemic Stroke With Regression Discontinuity Designs

**DOI:** 10.3389/fneur.2020.00961

**Published:** 2020-09-02

**Authors:** Andrew M. Naidech, Patrick N. Lawlor, Haolin Xu, Gregg C. Fonarow, Ying Xian, Eric E. Smith, Lee Schwamm, Roland Matsouaka, Shyam Prabhakaran, Ioana Marinescu, Konrad P. Kording

**Affiliations:** ^1^Department of Neurology, Northwestern University, Chicago, IL, United States; ^2^Division of Neurology, Children's Hospital of Philadelphia, Philadelphia, PA, United States; ^3^Duke Clinical Research Institute, Duke University, Durham, NC, United States; ^4^Division of Cardiology, University of California, Los Angeles, Los Angeles, CA, United States; ^5^Department of Neurology, Duke University Medical Center, Durham, NC, United States; ^6^Department of Neurology, University of Calgary, Calgary, AB, Canada; ^7^Department of Neurology, Massachusetts General Hospital, Boston, MA, United States; ^8^Department of Biostatistics and Bioinformatics, Duke University, Durham, NC, United States; ^9^Program for Comparative Effectiveness Methodology, Duke Clinical Research Institute, Duke University, Durham, NC, United States; ^10^School of Social Policy & Practice, University of Pennsylvania, Philadelphia, PA, United States; ^11^Departments of Neuroscience and Bioengineering, University of Pennsylvania, Philadelphia, PA, United States

**Keywords:** alteplase, ischemic stroke, regression discontinuity design, causal inference, quasi-experiments

## Abstract

Randomized Controlled Trials (RCTs) are considered the gold standard for measuring the efficacy of medical interventions. However, RCTs are expensive, and use a limited population. Techniques to estimate the effects of stroke interventions from observational data that minimize confounding would be useful. We used regression discontinuity design (RDD), a technique well-established in economics, on the Get With The Guidelines-Stroke (GWTG-Stroke) data set. RDD, based on regression, measures the occurrence of a discontinuity in an outcome (e.g., odds of home discharge) as a function of an intervention (e.g., alteplase) that becomes significantly more likely when crossing the threshold of a continuous variable that determines that intervention (e.g., time from symptom onset, since alteplase is only given if symptom onset is less than e.g., 3 h). The technique assumes that patients near either side of a threshold (e.g., 2.99 and 3.01 h from symptom onset) are indistinguishable other than the use of the treatment. We compared outcomes of patients whose estimated onset to treatment time fell on either side of the treatment threshold for three cohorts of patients in the GWTG-Stroke data set. This data set spanned three different treatment thresholds for alteplase (3 h, 2003–2007, *N* = 1,869; 3 h, 2009–2016, *N* = 13,086, and 4.5 h, 2009–2016, *N* = 6,550). Patient demographic characteristics were overall similar across the treatment thresholds. We did not find evidence of a discontinuity in clinical outcome at any treatment threshold attributable to alteplase. Potential reasons for failing to find an effect include violation of some RDD assumptions in clinical care, large sample sizes required, or already-well-chosen treatment threshold.

## Introduction

Randomized controlled trials (RCTs) are considered the gold standard in clinical investigation because, ideally, RCTs remove both known and unknown imbalances in groups that could lead investigators to wrongly conclude a treatment is efficacious (1). Data from RCTs are generally required before regulatory approval is granted to market a drug treatment (e.g., alteplase for acute ischemic stroke) (2), and new interventions typically require data from RCTs on efficacy before they are widely accepted.

Therapies that have a treatment threshold lead to challenging problems about the choice of those thresholds. In the case of alteplase, only patients whose symptoms began before the threshold time (e.g., 3 h prior to presentation) are eligible for treatment. The time window curtails treatment in clinical practice, and off-label use beyond approved time windows introduces legal and ethical concerns. If a treatment is effective within a narrow time window (e.g., 3 h for alteplase), there is typically a desire to extend it further to increase the number of patients who might be treated. Yet, each new time window typically requires another RCT, with the associated time and expenses of planning and conducting the trial. In the case of alteplase, data from other clinical trials was utilized to propose additional RCTs. However, extending the time window for fibrinolytic treatment expressly carried an increased risk of intracranial hemorrhage, which was borne out in a RCT with an extended time window (3). The concern for symptomatic hemorrhage has guided the design and conduct of RCTs for ischemic stroke generally (4, 5). Publication of an RCT showing that treatment up to 4.5 h after symptom onset was efficacious required several more years (6). Clinicians are often hesitant to wait years for new RCTs, and may treat patients outside of rigorously applied clinical trial protocols (7). Each new therapy (e.g., endovascular therapy for large vessel occlusion) brings a similar invitation to extend the window as long as it is efficacious. Conversely, some RCTs of time-limited therapies are negative, leading to the testing of more stringent time windows in hopes of finding efficacy [e.g., shortening the window of recombinant Factor VII for intracerebral hemorrhage from 3 h of symptom onset (8) to 2.5 h (9)]. Methods to hasten the determination of effective time windows for treatments with a threshold are needed.

New analytic techniques may improve our ability to determine the optimal treatment window for time-limited treatments. Regression Discontinuity Design (RDD), well-validated in economics and epidemiology (10–13), could be particularly helpful for determining if treatment thresholds are correctly set. RDD uses *observational data* to examine whether patients just above and just below the treatment threshold have different outcomes. RDD depends on the assumption that patients within a small window on either side of a threshold are no different other than being eligible for a treatment. We hypothesized that RDD would be a useful technique to evaluate alteplase treatment thresholds using observational data (clinical data), and that we could compare the results with those from already-conducted RCTs (6). RDD could eventually supplement RCTs in clinical decision making.

## Methods

We queried the Get With The Guidelines-Stroke (GWTG-Stroke) stroke data set, a long-standing, observational registry of patients with stroke. The methods of the GTWG-Stroke data set have been previously described in detail (14–16). To perform an RDD analysis, one requires for all patients the outcome variable of interest (e.g., home discharge), the continuous variable which determines the intervention (e.g., time from symptom onset until treatment decision), and the threshold value for this continuous variable that determines treatment administration [e.g., 3 h as shown in the original NINDS trial (2), or 4.5 h as in the ECASS III trial (6)]. If there is a difference (“discontinuity”) in outcomes between these two groups (that is not attributable to confounding variables), the difference in outcomes can be attributed to the treatment. To perform this analysis, a regression model is fit, including a parameter that estimates the magnitude of discontinuity in outcomes between patients on either side of the threshold.

We examined the following clinical outcomes for signs of a discontinuity: good discharge disposition, in-hospital mortality, length of hospital stay, ambulatory status at discharge, and modified Rankin Scale at discharge (see Supplementary Table 1 for details). We also analyzed factors that might have influenced the use or effectiveness of alteplase by building more sophisticated, adjusted models. These characteristics included patient age, sex, race/ethnicity, relevant past medical history (e.g., prior ischemic stroke, coronary heart disease, etc., NIH Stroke Scale (NIHSS), as well as hospital characteristics such as setting (urban, rural), and annual volume of patients with ischemic stroke. These demographic variables allow us to more accurately estimate the effect of alteplase, but also to confirm the validity of our model. The unadjusted models do not include this additional information. Since patients are assumed to be similar on either side of the threshold, a discontinuity in a demographic variable at the threshold could be evidence that this assumption is violated. Thus, we checked for discontinuities in demographic factors as well as in outcomes.

All participating institutions were required to comply with local regulatory and privacy guidelines and, if required, to secure institutional review board approval. Because data were used primarily at the local site for quality improvement, sites were granted a waiver of informed consent under the common rule.

## Methods: Running Variable Imputation

Our analysis required modification of the standard RDD setup due to different data recorded for treated and untreated patients. In a standard RDD, the running variable that determines the treatment is observed for both patients that are treated, as well as for patients that are not treated (e.g., a test score that determines a scholarship award). In our case, alteplase should only be administered within the time window in question (e.g., before 3 h) starting with the time of symptoms onset. Thus, for patients who are treated, the sequence of events that must take place within this window are: (1) development of symptoms, (2) clinical imaging (CT scan) to determine the need for treatment, and (3) the treatment itself. A reasonable running variable for this situation is the sum of these three times, which we call *onset to treatment time*. The situation for patients who are not treated is different because there is no time of treatment with alteplase. To address this issue, for both treated and untreated patients, we imputed the time from clinical imaging to treatment. We imputed this value using the median from the patient's hospital in that particular year. We call this running variable *estimated onset to treatment time* (OTT_*est*_). Multilevel modeling could be useful in such a scenario, but this is not yet routinely done in RDD analyses.

This method of estimating times for treated and untreated patients is reasonable. First, the time to administration of alteplase is known for each hospital each year, and reflects established protocols of stroke care. Second, the magnitude of the imputed value is about 20% of the total running variable; modest compared with the other two components. Third, the imputed *onset to treatment time* still reflects the most important clinical information that is used to determine alteplase administration (time from symptom development, and time of clinical imaging). We feel that the *onset to treatment time* and *estimated onset to treatment time* both contain essentially the same clinical information for treated and untreated patients, making it a reasonable running variable. Last, imputation of this value does not bias us toward finding an effect of alteplase, as would be the case if imputation introduced an artificial discontinuity in *estimated onset to treatment time*. Notably, the imputed time was used for both treated and untreated patients. Furthermore, *time from symptom development* and *time to imaging* for untreated patients are noisy measurements, and imputation of the *time from imaging to treatment* simply adds a fixed value to this. If anything, it introduces more noise, biasing us against finding a causal effect of alteplase.

## Methods: Sharp RDD

To determine if there is a discontinuity in outcome at the desired treatment threshold (e.g., 3 h from symptom onset), we constructed regression models that allow clinical outcomes (e.g., favorable hospital disposition) to be explained by the time from symptom onset to treatment—whether the patient was treated with alteplase—and patient- and hospital-specific characteristics (Equation 1). In the simplest form of RDD, sharp RDD, all patients on the left of the threshold (e.g., before 3 h) would receive alteplase, and none of the patients on the right of the threshold (e.g., after 3 h) would receive it. Treatment with alteplase is thus modeled as binary, and depends only on *estimated onset to treatment time* and the treatment threshold. Its corresponding regression parameter (Equation 1, parameter *b*_3_) is an estimate of the effect of treatment with alteplase. If there is a discontinuity in outcome at the treatment threshold (that is not attributable to confounding factors), the parameter relating alteplase administration to clinical outcome will be significantly different from zero.

We fit a logistic regression model of the log odds of favorable clinical outcome (a dichotomous variable, see Supplementary Table 1 for clinical outcomes examined), as a function of regressors given below, as well as patient- and hospital-specific characteristics (see Supplementary Table 2). NIH Stroke Scale was considered in a separate adjusted model due to a substantial amount of missing data (Supplementary Table 7). The adjusted equation for sharp RDD was:

(1) $$\begin{array}{l} \begin{array}{c} \ln\;\left(\frac{P\left(Y\right)}{1-P\left(Y\right)}\right)=LO\left(Y\right)=\\ b_{0}+b_{1}\left(\text{OT}{\text{T}}_{est}-c\right)+b_{2}\left(\text{OT}{\text{T}}_{est}-c\right)I\left[\text{OT}{\text{T}}_{est}\leq c\right]\\ +b_{3}I\left[\text{OT}{\text{T}}_{est}\leq c\right]+b_{4}X+\varepsilon \end{array} \end{array}$$

*Y* is the dichotomized clinical outcome, *P*(*Y*) is the probability of that outcome, and *LO*(*Y*) is the log odds of that outcome. OTT_*est*_ indicates *estimated onset to treatment time*; *I*[OTT_*est*_ ≤ *c*] is an indicator variable that equals 1 when the patient's onset to treatment time is less than the threshold time, *c*, and 0 otherwise; *c* has a value of 3 or 4.5 h depending on the cohort; *X* is a matrix containing the patient- and hospital-specific factors considered (see Supplementary Table 2); ε is an error term. The parameter *b*_3_ is the one of greatest interest, as it models the effect of alteplase treatment on log odds of clinical outcome at the threshold. The meaning of *b*_3_ can be seen by taking the difference in log odds of outcome of treated and untreated patients at the threshold (Equation 2); it is exactly the difference in log odds of outcome at the threshold. Exponentiating *b*_3_ gives the odds ratio of good clinical outcome to poor clinical outcome at the threshold. Values of exp(*b*_3_) greater than one (equivalent to *b*_3_ > 0) should be interpreted as increasing the odds of the clinical outcome of interest.

(2) $$\begin{array}{l} LO\left(Y\right){|}_{OTT=c_{-}}-LO\left(Y\right){|}_{OTT=c_{+}}=b_{3} \end{array}$$

The parameter *b*_0_ is an intercept term; *b*_1_ gives the slope of log odds of *Y* as a function of OTT_*est*_ on the right side of the cutoff; *b*_2_ is the parameter of the interaction term (OTT_*est*_ − *c*)*I*[OTT_*est*_ ≤ *c*], which effectively allows the slope to be different on the left and right sides of the threshold; *b*_4_ is the set of weights corresponding to each of the patient- and hospital-specific variables contained in *X*. In RDD analyses, the intercept and slope parameters are not typically of primary interest for causal interpretation. One can still, for example, speculate about the meaning of the slope, with the caveat that patients become less similar as you get farther from the threshold, and so are less comparable. In contrast, the discontinuity parameter, *b*_3_, is interpreted as the causal effect of the treatment at the threshold.

## Methods: Fuzzy RDD

In some cases, alteplase was administered outside of the treatment window, or not administered within the treatment window, meaning that the assumptions of sharp RDD do not strictly apply. Thus, we also applied fuzzy RDD, a technique used to model imperfect treatment compliance. Fuzzy RDD is widely used in health policy research and elsewhere (17, 18).

We used a two-stage, instrumental variable approach. Stage one models alteplase treatment administration (i.e., compliance); stage two models the clinical outcome as a function of OTT_*est*_ and alteplase administration, similar to sharp RDD. Both stages also include patient- and hospital-specific characteristics, as these could differentially affect alteplase administration and clinical outcome.

We modeled the log odds of alteplase administration as a function of running variable, treatment window threshold, and patient- and hospital-specific factors. The equation for Stage 1 was:

(3) $$\begin{array}{l} \ln\;\left(\frac{P\left(\text{tPA}\right)}{1-P\left(\text{tPA}\right)}\right)\text{=}LO\left(\text{tPA}\right)\text{=}b_{0}^{\left(1\right)}+b_{1}^{\left(1\right)}I\left[\text{OT}{\text{T}}_{est}\leq c\right]\\ \;+b_{2}^{\left(1\right)}X+\varepsilon \end{array}$$

Here, *P*(tPA) is the probability of alteplase administration, and *LO*(tPA) is the log odds of tPA administration. OTT_*est*_ indicates the imputed onset to treatment time. The parameters of this equation, $\left\{b_{i}^{\left(1\right)}\right\}$ have a superscript that denotes the stage of the model (first stage, here), and a subscript that uniquely identifies each term. The parameter $b_{0}^{\left(1\right)}$ is an intercept for the model of alteplase compliance; $b_{1}^{\left(1\right)}$ is a parameter that models alteplase compliance on the left side of the threshold; $b_{2}^{\left(1\right)}$ is a set of parameters that models alteplase administration as a function of patient- and hospital-specific factors.

In the second stage, we modeled the log odds of clinical outcome as a function of running variable, treatment window threshold, and patient- and hospital- specific factors. The equation for Stage 2 was:

(4) $$\begin{array}{l} \;\ln\;\left(\frac{P\left(Y\right)}{1-P\left(Y\right)}\right)=b_{0}^{\left(2\right)}+b_{1}^{\left(2\right)}\left(\text{OT}{\text{T}}_{est}-c\right)\\ +b_{2}^{\left(2\right)}\left(\text{OT}{\text{T}}_{est}-c\right)LO\left(\text{tPA}\right)+b_{3}^{\left(2\right)}LO\left(\text{tPA}\right)+b_{4}^{\left(2\right)}X+\varepsilon \end{array}$$

Here, again, *Y* refers to the dichotomized clinical outcome and *P*(*Y*)to the probability of the clinical outcome. *LO*(tPA)refers to the predicted log odds of alteplase administration, from the first stage (Equation 3). The parameters of this equation, $\left\{b_{i}^{\left(2\right)}\right\}$have a superscript that denotes the stage of the model (second stage, here), and a subscript that uniquely identifies each term. The parameter $b_{3}^{\left(2\right)}$ is again of greatest interest, as it models the effect of alteplase treatment on log odds of clinical outcome. The meaning of $b_{3}^{\left(2\right)}$ can be seen by taking the difference in log odds of outcome of treated and untreated patients at the threshold (Equation 5). Unlike sharp RDD, the difference in log odds depends on $b_{3}^{\left(2\right)}$ as well as $b_{1}^{\left(1\right)}$. This makes sense because $b_{1}^{\left(1\right)}$ is the additional log odds of alteplase administration for patients with OTT_*est*_ within the treatment window. In other words, outcomes in a fuzzy RDD model depend both on the treatment effect size and the compliance. Values of $b_{3}^{\left(2\right)}>0$ should be interpreted as increasing the odds of the clinical outcome of interest.

(5) $$\begin{array}{l} LO\left(Y\right){|}_{OTT=c_{-}}-LO\left(Y\right){|}_{OTT=c_{+}}=b_{3}^{\left(2\right)}b_{1}^{\left(1\right)} \end{array}$$

The parameter $b_{0}^{\left(2\right)}$ is an intercept term; $b_{1}^{\left(2\right)}$ gives the slope of log odds of *Y* as a function of OTT_*est*_ independent of alteplase administration (analogous to *b*_1_ in Equation 1); $b_{2}^{\left(2\right)}$ gives the slow of log odds of *Y* as a function of (OTT_*est*_ − *c*)*LO*(tPA) (analogous to *b*_2_ in Equation 1); *b*_4_ is the set of weights corresponding to each of the patient- and hospital-specific variables contained in *X*.

Another important issue with instrumental variable models is the “strength” of the instrument. If there were a weak or non-existent relationship between *estimated onset to treatment time* and *odds of tPA administration*, there would be no point in examining whether *estimated onset to treatment time* affects clinical outcomes, since our proposed model is that *estimated onset to treatment time* affects *odds of alteplase administration* which in turn may affect *clinical outcome*. Supplementary Table 3 shows percentage of patients receiving alteplase on each side of the threshold in each cohort. In all cohorts there is meaningful difference in odds of alteplase administration. Thus, we have a relatively strong instrument to examine the effect of alteplase on clinical outcomes.

Analyses were performed using the SAS version 9.4 (SAS Institute Inc., Cary, NC, USA) and R version 3.4.4 (R Core Team, 2018, Vienna, Austria). All *P*-values are 2-sided tests and were considered statistically significant at <0.05. Duke Clinical Research Institute (DCRI) served as the data analysis center.

## Results

We analyzed three separate cohorts within the GWTG-Stroke registry (Table 1). Cohort A includes patients prior to 2008, when 3 h was the accepted treatment threshold (Table 2). Calendar year 2008 was not modeled, as this was the year that ECASS III reported alteplase was effective up to 4.5 h after symptom onset (6), which represented a new treatment threshold, and practice shifted. In 2009 and afterwards, treatment thresholds of both 3 and 4.5 h were observed for ongoing study. Cohort B includes patients after 2009 who were treated according to the 4.5 h threshold (Table 3). Cohort C includes patients after 2009 who were treated according to the 3 h threshold (Table 4). We included only patients with *estimated onset to treatment* times within 20 min of the treatment threshold, with valid imaging, and valid imaging times. We excluded inter-hospital transfers, stroke occurring after hospital arrival, contraindications to alteplase, and enrollment in clinical trials of alteplase. After these restrictions, there were 1,869 patients for analysis in Cohort A, 6,550 patients in Cohort B, and 13,086 patients in Cohort C. Cohorts on either side of the treatment threshold were generally well-matched, although there were some differences (Supplementary Tables 4–6; and see below).

**Table 1:** Cohort Demographics.

**Variable**	**Cohort A (2.67–3.3 h, <2009)**	**Cohort B (4.16–4.83 h, >2009)**	**Cohort C (2.67–3.3 h, >2009)**
N	1,869	6,550	13,086
Age, years	73 (61–82)	73 (61–83)	72 (61.8)
Race, White	77.7%	71.5%	71.9%
Black	12.2%	15.5%	14.3%
Asian	1.8%	2.4%	7.0%
Other	3.9%	3.5%	3.8%
NIH Stroke Scale	9 (5–15)	5 (2–11)	8 (4–14)
Women	48.7%	50.2%	50.4%
Atrial fibrillation	22.1%	19.1%	19.7%
Prior Stroke or TIA	27.8%	31.1%	27.6%
Coronary artery disease	29.4%	26.2%	25.1%
Diabetes mellitus	28.7%	31.7%	29.7%
Historical hypertension	72.6%	76.3%	74.2%
Tobacco Use	19.8%	17.5%	17.2%
Dyslipidemia	35.9%	45.3%	43.9%
Primary Stroke Center	44.8%	46.4%	45.9%
Academic Hospital	66.6%	61.8%	63.1%

*Data are N, %, or mean (25–75%ile)*.

**Table 2:** RDD results from Cohort A (treatment threshold of 3 h, prior to 2008).

**Outcomes**	**Coefficients**	**Unadjusted**		**Adjusted**		**NIHSS Adjusted**	
**Sharp RDD**	**Discontinuity, **exp(*b*_3_)****	**OR (95% CI)**	***P*-value**	**OR (95% CI)**	***P*-value**	**OR (95% CI)**	***P*-value**
Good discharge disposition	Before vs. after	0.91 (0.63–1.32)	0.621	0.88 (0.57–1.36)	0.570	0.78 (0.44–1.38)	0.394
Discharged home	Before vs. after	0.89 (0.63–1.24)	0.480	0.86 (0.60–1.24)	0.424	0.91 (0.53–1.54)	0.718
In-hospital mortality	Before vs. after	0.86 (0.46–1.61)	0.644	0.87 (0.46–1.64)	0.669	0.69 (0.29–1.62)	0.390
LOS ≤ 4 days	Before vs. after	0.71 (0.51–1.00)	0.052	0.70 (0.49–1.00)	0.048	0.85 (0.54–1.33)	0.469
Ambulate independently at discharge	Before vs. after	0.90 (0.63–1.28)	0.550	0.85 (0.58–1.26)	0.429	0.90 (0.52–1.55)	0.696
mRS 0–1 vs. 2–6	Before vs. after	–		–		–	
**Fuzzy RDD**		**Estimate**	***P*****-value**	**Estimate**	***P*****-value**	**Estimate**	***P*****-value**
**Stage 1**	$b_{1}^{\left(1\right)}$						
tPA	Before vs. after cut-off	1.055	<.0001	1.113	<.0001	1.177	<.0001
**Stage 2**	$b_{3}^{\left(2\right)}$						
Good disposition	tPA Yes/No	−0.366	0.621	−0.764	0.357	−1.879	0.074
Discharged home	tPA Yes/No	−0.468	0.480	−0.668	0.362	−0.867	0.398
In-hospital mortality	tPA Yes/No	−0.574	0.644	−0.085	0.949	0.328	0.851
LOS ≤ 4 days	tPA Yes/No	−1.312	0.052	−1.460	0.035	−1.021	0.230
Able to ambulate independently at discharge	tPA Yes/No	−0.417	0.550	−1.100	0.158	−1.133	0.289
mRS 0–1 vs. 2–6	tPA Yes/No	–		–		–	

**Table 3:** RDD results from Cohort B (treatment threshold of 4.5 h, after 2009).

**Outcomes**	**Coefficients**	**Unadjusted**		**Adjusted**		**NIHSS Adjusted**	
**Sharp RDD**	**Discontinuity, **exp(*b*_3_)****	**OR (95% CI)**	***P*-value**	**OR (95% CI)**	***P*-value**	**OR (95% CI)**	***P*-value**
Good discharge disposition	Before vs. after	0.95 (0.76–1.18)	0.627	0.92 (0.72–1.17)	0.504	0.86 (0.64–1.15)	0.300
Discharged home	Before vs. after	1.13 (0.93–1.37)	0.230	1.11 (0.90–1.38)	0.312	1.20 (0.92–1.55)	0.171
In-hospital mortality	Before vs. after	1.09 (0.70–1.71)	0.701	1.12 (0.71–1.77)	0.637	1.27 (0.71–2.28)	0.417
LOS ≤ 4 days	Before vs. after	1.21 (1.00–1.47)	0.056	1.20 (0.98–1.46)	0.081	1.33 (1.05–1.67)	0.017
Able to ambulate independently at discharge	Before vs. after	0.97 (0.80–1.19)	0.790	0.95 (0.77–1.17)	0.615	0.92 (0.72–1.18)	0.498
mRS 0–1 vs. 2–6	Before vs. after	1.26 (0.85–1.88)	0.254	1.29 (0.84–2.00)	0.250	1.42 (0.89–2.28)	0.144
**Fuzzy RDD**		**Estimate**	***P*****-value**	**Estimate**	***P*****-value**	**Estimate**	***P*****-value**
**Stage 1**	$b_{1}^{\left(1\right)}$						
tPA	Before vs. after cutoff	0.977	<.0001	1.013	<.0001	1.043	<.0001
**Stage 2**	${\text{b}}_{3}^{\left(2\right)}$						
Good discharge disposition	tPA Yes/No	−0.304	0.627	−0.517	0.408	−0.452	0.482
Discharged home	tPA Yes/No	0.653	0.230	0.482	0.353	1.292	0.028
In-hospital mortality	tPA Yes/No	0.484	0.701	0.071	0.951	0.652	0.579
LOS ≤ 4 days	tPA Yes/No	1.051	0.056	0.859	0.073	1.072	0.031
Able to ambulate independently at discharge	tPA Yes/No	−0.150	0.790	−0.543	0.304	−0.124	0.827
mRS 0–1 vs. 2–6	tPA Yes/No	1.276	0.254	0.265	0.791	1.037	0.291

**Table 4:** RDD results from Cohort C (treatment threshold of 3 h, after 2009).

**Outcomes**	**Coefficients**	**Unadjusted**		**Adjusted**		**NIHSS Adjusted**	
**Sharp RDD**	**Discontinuity, **exp(*b*_3_)****	**OR (95% CI)**	***P*-value**	**OR (95% CI)**	***P*-value**	**OR (95% CI)**	***P*-value**
Good discharge disposition	Before vs. after	1.00 (0.86–1.17)	0.986	1.07 (0.90–1.26)	0.442	0.98 (0.81–1.19)	0.850
Discharged home	Before vs. after	1.00 (0.88–1.15)	0.948	1.06 (0.91–1.24)	0.431	1.04 (0.87–1.23)	0.691
In-hospital mortality	Before vs. after	1.07 (0.78–1.45)	0.681	1.04 (0.77–1.40)	0.795	1.22 (0.87–1.71)	0.246
LOS ≤ 4 days	Before vs. after	1.04 (0.90–1.19)	0.601	1.06 (0.91–1.22)	0.462	1.02 (0.87–1.19)	0.830
Able to ambulate independently at discharge	Before vs. after	0.92 (0.81–1.06)	0.252	0.96 (0.83–1.11)	0.588	0.91 (0.78–1.06)	0.237
mRS 0–1 vs. 2–6	Before vs. after	0.96 (0.74–1.24)	0.739	0.98 (0.75–1.29)	0.902	0.95 (0.71–1.28)	0.747
**Fuzzy RDD**		**Estimate**	***P*****-value**	**Estimate**	***P*****-value**	**Estimate**	***P*****-value**
**Stage 1**	$b_{1}^{\left(1\right)}$						
tPA	Before vs. after	0.723	<.0001	0.756	<.0001	0.858	<.0001
**Stage 2**	$b_{3}^{\left(2\right)}$						
Good discharge disposition	tPA Yes/No	0.012	0.986	−0.266	0.687	−0.864	0.125
Discharged home	tPA Yes/No	0.039	0.948	−0.204	0.736	−1.193	0.019
In-hospital mortality	tPA Yes/No	0.557	0.681	1.166	0.327	1.830	0.153
LOS ≤ 4 days	tPA Yes/No	0.315	0.601	0.260	0.647	−1.067	0.021
Able to ambulate independently at discharge	tPA Yes/No	−0.674	0.252	−0.952	0.107	−1.534	0.001
mRS 0–1 vs. 2–6	tPA Yes/No	−0.376	0.739	−1.232	0.260	−2.267	0.009

The central result of our study was that, in terms of clinical outcomes (e.g., odds of good discharge disposition), there was no strong evidence of a discontinuity at either the 3 or 4.5 h threshold. This was true using both sharp and fuzzy RDD, suggesting that the result does not depend strongly on the formulation of the model. This was also true using both simple models without patient- and hospital-specific factors and NIH stroke scale, as well as models with those factors. This suggests that the result does not depend strongly on model complexity, and is not related to changes in patient characteristics across the alteplase administration threshold.

We did detect some weak effects with this analysis, however. In Cohort A, the odds of length of stay <4 days was significantly different across the threshold in both the sharp and fuzzy adjusted models (Table 2). But the direction of the effect was opposite what would be expected; patients treated with alteplase were less likely to have a length of stay <4 days than were the untreated patients. In Cohorts B and C, there were multiple outcomes with significant differences across the threshold in the NIHSS-adjusted models (Tables 3, 4). Some of these effects, again, had the opposite direction that would be expected with alteplase treatment (negative values of $b_{3}^{\left(2\right)}$). Furthermore, there was a significant amount of missing NIHSS data (Supplementary Table 7), and likely as a result, there were significant differences in initial NIHSS score across the thresholds (see below). Overall, we feel that these results are more likely explained by the fact that we tested many outcomes and would expect some to be positive by chance, and by biased subgroups. We thus do not feel justified in claiming any strong effects of alteplase administration at the treatment thresholds of 3 or 4.5 h.

We also checked for confounding by examining whether patient- and hospital-specific quantities (e.g., demographic factors) had discontinuities at the treatment thresholds. For an RDD to be valid, there should not be discontinuities in these quantities at the threshold, since any putative discontinuity in outcome could be attributed to the discontinuity in these factors rather than to the treatment itself. Thus, this analysis would be more important if there were significant differences in clinical outcome, which we did not find. Nevertheless, we tested many patient- and hospital-specific quantities (Supplementary Tables 4–6). Cohorts B and C included significantly more patients than Cohort A, and had more significant differences in patient- and hospital- specific quantities across the threshold. In cohorts B and C, initial NIHSS was higher in post-threshold patients than pre-threshold patients. NIHSS was missing in a larger percentage of pre-threshold patients in Cohorts A and C. This likely means that NIHSS score measurement was not random, and that the NIHSS-possessing subgroups were not as comparable on either side of the treatment thresholds (see paragraph above). This casts some doubt on the results of the NIHSS-adjusted models. Ambulatory status was different across the threshold in Cohorts B and C. Overall, there were some patient- and hospital-specific quantities that were imbalanced across the threshold. Some of this may be due to true imbalance, some due to chance, and perhaps some due to differences in data collection on each side of the threshold, which is likely not as consistent as in an RCT. Again, the lack of differences in outcome make this analysis less crucial. Experimental techniques short of experimental randomization do have their limitations, but it is also important to keep in mind that RCTs can also have such imbalance that does not necessarily threaten causal validity.

## Discussion

Using RDD in a nationwide data set, we found no convincing evidence of a discontinuity in the effectiveness of alteplase on clinical outcomes after ischemic stroke around the 3 and 4.5 h treatment thresholds. This result is not necessarily inconsistent with an RCT (6) showing positive, but modest, effects in the 3 to 4.5 h window. These data suggest that using RDD to extend treatment windows for alteplase may be challenging.

There are several potential explanations for not detecting an effect of alteplase at these thresholds. First, our analyses sought to detect a difference during the hospitalization or at the time of hospital discharge. Alteplase leads to improved outcome at 3 months, not to improved discharge disposition, so functional outcomes [e.g., the modified Rankin Scale (2, 6) months later] or health-related quality of life (19–21), might be more likely to show effects of alteplase treatment. Next, our study may have been underpowered. We chose a window of 20 min on either side of the treatment threshold, which led to smaller cohorts. These potential limitations may have attenuated our ability to detect an effect using RDD. Next, our continuous variable, *estimated onset to treatment time*, was imputed using hospital-specific characteristics, which added some noise near the threshold, further reducing power. Lastly, treatment thresholds chosen by expert clinicians may be accurate guesses of the optimal treatment threshold, and so further extensions of time windows for treatment may have small marginal benefits (22, 23). For example, if patients just outside the treatment threshold could benefit from alteplase, one would expect the difference in outcomes at the threshold to be larger. Thresholds chosen by experts may also incorporate other information that affects outcome, such as the increased risk of intracerebral hemorrhage due to alteplase, which increases with an expanded time window from symptom onset to treatment (3). These potential limitations may have attenuated our ability to detect an effect using RDD. Future research using RDD will need to carefully choose questions where there are substantial existing data that closely approximate the clinical trial question of interest.

In summary, our RDD analysis did not find a discontinuity in disposition around 3 or 4.5 h alteplase treatment thresholds in acute ischemic stroke. Future investigations with RDD might leverage large data sets with outcome measures sensitive to the intervention, that can be culled from the electronic health record, potentially combining multiple hospitals and institutions.

## Data Availability Statement

The datasets generated for this study will not be made publicly available. Data are maintained by the Get With The Guidelines (GWTG) consortium at the Duke Clinical Research Institute, where the analysis was performed. Authors should contact GWTG with data requests.

## Ethics Statement

The DUHS IRB has determined the specific components in this study to be in compliance with all applicable Health Insurance Portability and Accountability Act (HIPAA) regulations. The DUHS IRB waived the need for formal ethical approval for this study. Written informed consent was not required as per local legislation.

## Author Contributions

AN introduced the concept with KK and co-wrote the manuscript. PL co-wrote the manuscript. HX, YX, and RM performed and oversaw statistical analysis and edited the manuscript. GF, ES, LS, and RM revised the manuscript for intellectual content. SP developed the concept with AN and KK and revised the manuscript. IM revised the manuscript for intellectual content and oversaw the statistical analysis. KK developed the concept and revised the manuscript for intellectual content. All authors contributed to the article and approved the submitted version.

## Conflict of Interest

GF: research support from PCORI and NIH, and an employee of University of California which holds a patent on an endovascular device for stroke. AN: research support from NIH and AHRQ. SP: research support from PCORI and NIH. KK: research support from NIH. LS: reports being the principal investigator of an investigator-initiated study of extended-window intravenous thrombolysis funded by the National Institutes of Neurological Disorders and Stroke (clinicaltrials.gov/show/NCT01282242) for which Genentech provided alteplase free of charge to Massachusetts General Hospital as well as supplemental per-patient payments to participating sites; serving as chair of the AHA/ASA GWTG stroke clinical work group and Quality Oversight Committees, serving as a stroke systems consultant to the Massachusetts Department of Public Health; and serving as a scientific consultant regarding trial design and conduct to Penumbra (data and safety monitoring committee, Separator 3D and MIND trials) and Medtronic (Victory AF and Stroke AF trials). The remaining authors declare that the research was conducted in the absence of any commercial or financial relationships that could be construed as a potential conflict of interest.
