# Supplementary material for: Probing the Effective Treatment Thresholds for Alteplase in Acute Ischemic Stroke With Regression Discontinuity Designs
Source: Front Neurol. 2020 Sep 2;11:961. doi: 10.3389/fneur.2020.00961 (PMC7492202; doi:10.3389/fneur.2020.00961)
Supplement: Supplementary file 1 [file Data_Sheet_1.pdf]

Supplementary Tables.

Supplementary Table 1. Clinical Outcomes Variables

Outcome variable	Possible values	Dichotomized levels
Discharge disposition	Home; hospice; skilled nursing facility; inpatient rehabilitation facility; other facilities; death	Good disposition (home, inpatient rehabilitation facility); poor disposition (hospice, skilled nursing facility, other facilities, death)
In-hospital mortality	Yes; no	Yes; no
Length of stay	Number of days	Short length of stay (<=4 days); long length of stay (>4 days)
Ambulatory status at discharge	Able to ambulate independently; able to ambulate with help from others; not able to ambulate; death	Good ambulatory status (able to ambulate independently); poor ambulatory status (not able to ambulate independently)
Modified Rankin Scale	0-6	Good (0-1); poor (2-6)

Supplementary Table 2. Patient and Hospital Characteristics

Patient demographic characteristics	Age, sex, race/ethnicity, insurance status
Patient medical history	Atrial fibrillation/flutter, prosthetic heart valve, prior stroke/transient ischemic attack, coronary artery disease/myocardial infarction, carotid stenosis, peripheral vascular disease, hypertension, smoker, dyslipidemia, heart failure
Hospital characteristics	Rural location, region, primary stroke center, academic status, number of beds, annual ischemic stroke volume, annual IV tPA volume

Supplementary 3. Treatment compliance

Variable	Cohort A	Cohort B	Cohort C
OTT times in the window of threshold ± 20min	1,869	6,550	13,086
% patients treated with tPA on left or on right of threshold	63.4% / 36.9%	34.6% / 15.7%	86.9% / 74.6%

Supplementary Table 4. Confounder analysis, Cohort A (treatment threshold of 3 hours, prior to 2008)

Variable	Overall N=1,869	20min before 3h N=762	20min after 3h N=1,107	P Value	Standardized Difference, %
**Demographics**					
Age	73 (61 - 82)	74 (62 - 82)	72 (60 - 81)	0.063	9.5
Female (%)	48.7	49.7	48.1	0.475	3.4
Race/Ethnicity (%)				0.641	
White	77.7	77.4	77.9		1.1
Black	12.2	11.4	12.7		4.1
Hispanic (any race)	4.4	4.6	4.3		1.2
Asian	1.8	2.1	1.6		3.5
Other (includes UTD)	3.9	4.5	3.4		5.3
Health Insurance Status (%)				0.126	
Medicare	4.9	5.4	4.5		5.8
Medicaid	50.4	52.2	49.3		35.6
Private/Other Insurance	3.5	0.0	6.0		4.3
Self-Pay/No Insurance	41.2	42.4	40.3		4.4
**Medical History**					
Atrial Fibrillation/Flutter (%)	22.1	22.6	21.8	0.678	2.0
Prosthetic Heart Valve (%)	1.3	1.3	1.4	0.938	0.4
Prior Stroke/TIA (%)	27.7	28.9	26.9	0.352	4.4
CAD/Prior MI (%)	29.4	30.5	28.6	0.372	4.2
Carotid Stenosis (%)	3.6	4.1	3.3	0.409	3.9
Diabetes Mellitus (%)	28.7	29.2	28.4	0.714	1.7

PVD (%)	4.3	5.0	3.9	0.249	5.4
Hypertension (%)	72.6	72.7	72.5	0.942	0.3
Smoker (%)	19.8	19.2	20.2	0.602	2.5
Dyslipidemia (%)	35.9	36.9	35.2	0.438	3.7
Heart Failure (%)	0.9	1.1	0.7	0.450	3.5
Medical History Panel Missing (%)	0.1	0.1	0.1	0.791	1.2
**Arrival Information**					
Arrival Mode: EMS (%)	80.6	79.4	81.4	0.284	5.0
Ambulatory Status at Admission (%)				0.480	
Ambulate independently	33.3	0.0	50.0		141.4
Need assistance from person	66.7	100.0	50.0		141.4
Unable to ambulate	0.0	0.0	0.0		0.0
Initial NIHSS Score (0-42)	9 (5 - 15)	9 (5 - 15)	10 (5 - 15)	0.104	7.9
NIHSS missing (%)	33.4	37.9	30.4	0.001	16.0
On-time Arrival (non-holiday weekday, 7am-6pm) (%)	45.7	44.4	46.7	0.317	4.7
**Hospital Characteristics**					
Rural Location (%)	1.9	2.0	1.9	0.912	0.5
Region (%)				0.845	
West	17.9	18.0	17.9		0.2
South	35.3	34.3	36.0		3.8
Midwest	19.6	20.3	19.1		3.2
Northeast	27.2	27.4	27.0		0.9
Primary Stroke Center (%)	44.9	44.8	45.1	0.889	0.7
Academic Hospital (%)	66.6	67.5	66.0	0.523	3.0

Number of Beds	425 (315 - 613)	439 (328 - 616)	424 (306 - 610)	0.083	8.8
Annual IS Admission Volume	273 (183 - 383)	284 (190 - 388)	265 (181 - 379)	0.089	7.2
Annual IV t-PA Volume	23 (14 - 32)	24 (14 - 32)	23 (13 - 34)	0.916	1.5

Supplementary Table 5. Confounder analysis, Cohort B (treatment threshold of 4.5 hours, after 2009)

Variable	Overall<br>N=6,550	20min before 4.5h<br>N=3,112	20min after 4.5h<br>N=3,438	P Value	Standardized Differences, %
**Demographics**					
Age	73 (61 - 83)	73 (62 - 84)	72 (61 - 83)	0.011	6.3
Female (%)	50.2	50.2	50.1	0.949	0.2
Race/Ethnicity (%)				0.857	
White	71.5	71.8	71.3		1.1
Black	15.5	15.2	15.7		1.6
Hispanic (any race)	7.1	7.3	6.9		1.4
Asian	2.4	2.4	2.4		0.4
Other (includes UTD)	3.5	3.3	3.7		1.9
Health Insurance Status (%)				0.037	
Medicare	5.9	5.6	6.2		7.6
Medicaid	38.6	40.6	36.8		0.8
Private/Other Insurance	10.1	9.9	10.2		5.6
Self-Pay/No Insurance	45.4	43.9	46.7		2.8
**Medical History**					
Atrial Fibrillation/Flutter (%)	19.1	19.7	18.6	0.260	2.8
Prosthetic Heart Valve (%)	1.4	1.0	1.9	0.003	7.5
Prior Stroke/TIA (%)	31.1	31.6	30.6	0.376	2.2
CAD/Prior MI (%)	26.2	26.9	25.6	0.238	2.9
Carotid Stenosis (%)	3.5	3.1	3.9	0.103	4.1
Diabetes Mellitus (%)	31.7	32.4	31.0	0.242	2.9

PVD (%)	4.6	4.5	4.7	0.784	0.7
Hypertension (%)	76.3	77.2	75.5	0.101	4.1
Smoker (%)	17.5	17.2	17.8	0.506	1.7
Dyslipidemia (%)	45.3	46.6	44.1	0.040	5.1
Heart Failure (%)	9.3	9.6	9.0	0.368	2.2
Medical History Panel Missing (%)	0.9	0.7	1.0	0.142	3.6
**Arrival Information**					
Arrival Mode: EMS (%)	65.0	64.3	65.6	0.263	2.8
Ambulatory Status at Admission (%)				0.033	
Ambulate independently	37.2	38.9	35.7		6.6
Need assistance from person	25.9	26.2	25.6		1.4
Unable to ambulate	36.9	35.0	38.7		7.8
Initial NIHSS Score (0-42)	5 (2 - 11)	5 (2 - 11)	6 (3 - 12)	0.0002	8.6
NIHSS missing (%)	22.1	22.5	21.8	0.454	1.9
On-time Arrival (non-holiday weekday, 7am-6pm) (%)	43.7	42.8	44.5	0.173	3.4
**Hospital Characteristics**					
Rural Location (%)	3.7	3.4	3.9	0.295	2.6
Region (%)				0.074	
West	18.9	18.2	19.6		3.7
South	38.0	37.3	38.7		3.0
Midwest	18.2	19.2	17.2		5.3
Northeast	24.9	25.3	24.5		1.9
Primary Stroke Center (%)	46.4	45.8	46.9	0.401	2.1
Academic Hospital (%)	61.8	61.2	62.4	0.314	2.5

Number of Beds	375 (261 - 555)	374 (258 - 550)	377 (263 - 556)	0.391	0.8
Annual IS Admission Volume	242 (165 - 353)	242 (168 - 353)	242 (162 - 349)	0.261	3.3
Annual IV t-PA Volume	20 (12 - 32)	20 (13 - 32)	20 (12 - 32)	0.626	0.0

Supplementary Table 6. Confounder analysis, Cohort C (treatment threshold of 3 hours, after 2009)

Variable	Overall N=13,086	20min before 3h N=5,429	20min after 3h N=7,657	P Value	Standardized Difference, %
**Demographics**					
Age	72 (61 - 83)	72 (60 - 82)	73 (61 - 83)	0.0004	6.2
Female (%)	50.4	50.3	50.5	0.867	0.3
Race/Ethnicity (%)				0.364	
White	71.9	71.3	72.3		2.2
Black	14.3	14.7	14.1		1.9
Hispanic (any race)	7.0	7.4	6.7		2.7
Asian	2.9	2.8	3.0		1.3
Other (includes UTD)	3.8	3.7	3.9		0.9
Health Insurance Status (%)				0.065	
Medicare	6.3	6.1	6.5		4.5
Medicaid	38.8	37.5	39.8		0.6
Private/Other Insurance	9.3	9.5	9.3		4.7
Self-Pay/No Insurance	45.5	46.9	44.5		1.3
**Medical History**					
Atrial Fibrillation/Flutter (%)	19.6	19.7	19.6	0.972	0.1
Prosthetic Heart Valve (%)	1.1	1.2	1.1	0.540	1.1
Prior Stroke/TIA (%)	27.6	27.6	27.7	0.890	0.2
CAD/Prior MI (%)	25.1	24.1	25.8	0.026	4.0
Carotid Stenosis (%)	2.8	2.9	2.8	0.791	0.5
Diabetes Mellitus (%)	29.7	29.4	29.9	0.521	1.1

PVD (%)	3.7	3.8	3.7	0.804	0.4
Hypertension (%)	74.2	73.2	75.0	0.022	4.1
Smoker (%)	17.2	17.8	16.8	0.149	2.6
Dyslipidemia (%)	43.9	43.9	43.9	0.970	0.1
Heart Failure (%)	9.0	9.1	8.9	0.624	0.9
Medical History Panel Missing (%)	0.7	0.8	0.6	0.305	1.8
**Arrival Information**					
Arrival Mode: EMS (%)	73.6	71.3	75.2	<.0001	8.9
Ambulatory Status at Admission (%)				0.0006	
Ambulate independently	25.8	28.0	24.2		8.7
Need assistance from person	22.7	22.3	22.9		1.5
Unable to ambulate	51.6	49.7	52.9		6.4
Initial NIHSS Score (0-42)	8 (4 - 14)	7 (4 - 14)	8 (5 - 14)	<.0001	5.9
NIHSS missing (%)	9.1	10.8	8.0	<.0001	9.6
On-time Arrival (non-holiday weekday, 7am-6pm) (%)	46.5	45.0	47.5	0.005	5.0
**Hospital Characteristics**					
Rural Location (%)	3.7	3.9	3.7	0.530	1.1
Region (%)				0.097	
West	20.5	20.8	20.3		1.1
South	36.3	35.6	36.8		2.4
Midwest	18.5	19.4	17.9		3.9
Northeast	24.6	24.2	25.0		1.9
Primary Stroke Center (%)	45.9	47.0	45.1	0.029	3.9
Academic Hospital (%)	63.1	63.2	63.0	0.785	0.5

Number of Beds	382 (265 - 560)	380 (263 - 555)	388 (266 - 572)	0.021	5.9
Annual IS Admission Volume	243 (169 - 372)	240 (168 - 363)	245 (169 - 376)	0.300	2.3
Annual IV t-PA Volume	23 (14 - 34)	22 (14 - 34)	24 (14 - 34)	0.023	4.0

Supplementary Table 7. Missing data in demographic variables

Variables Missing Rates (%)	Cohort A	Cohort B	Cohort C
**Patient Demographics** (%)			
Age*	0	0	
Female	0	0	
Race/Ethnicity	0	0	
Health Insurance Status	87.9	14.6	15.0
**Medical History** (%)	0.1	0.9	0.7
**Arrival Information** (%)			
Arrival Mode: EMS	1.4	5.4	5.6
Ambulatory Status at Admission	99.8	37.8	40.8
Initial NIHSS	33.4	22.1	9.1
**Hospital Characteristics** (%)	0	0	0
